# Cardiovascular consequences of aircraft noise exposure

**DOI:** 10.3389/fpubh.2022.1058423

**Published:** 2022-12-02

**Authors:** Justyna Ba̧czalska, Wiktoria Wojciechowska, Marta Rojek, Omar Hahad, Andreas Daiber, Thomas Münzel, Marek Rajzer

**Affiliations:** ^1^First Department of Cardiology, Interventional Electrocardiology and Arterial Hypertension, Jagiellonian University Medical College, Kraków, Poland; ^2^Department of Cardiology, University Medical Center Mainz, Johannes Gutenberg University, Mainz, Germany

**Keywords:** aircraft noise, environmental noise, noise exposure, cardiovascular diseases, hypertension

## Abstract

The results from epidemiological studies suggest that environmental noise including aircraft, railway, road traffic, wind turbine, and leisure-related noise is a growing public health concern. According to the WHO, at least 100 million people in the European Union are affected by traffic noise levels above the WHO-recommended thresholds. Environmental noise can adversely affect physical and mental health, as well as wellbeing. Chronic low-level noise exposure typical for most environmental sources is associated with psychophysiological stress causing non-auditory or indirect noise effects leading ultimately to cardiovascular diseases. Among all environmental noise sources, aircraft noise is considered the most annoying, and its leading mechanism of action is autonomic system activation such as increases in heart rate and blood pressure. Previously, we observed that long-term exposure to aircraft noise was associated with increased diastolic blood pressure, arterial stiffness (as assessed by pulse wave velocity), and impaired left ventricular diastolic function. All mentioned above effects are early, subclinical, and potentially reversible changes which preceded late noise effects in the cardiovascular system, that is, established cardiovascular diseases such as myocardial infarction, stroke, and heart failure. However, even a short-term reduction in aircraft noise exposure as observed during the COVID-19 lockdown may reverse these negative effects on arterial stiffness and blood pressure and may decrease the prevalence of insomnia. In this review, we aimed to critically discuss our obtained results considering recent studies on the influence of aircraft noise (and other traffic noises) on cardiovascular diseases in the context of the WHO Environmental Noise Guidelines for the European Region.

## Introduction

Cardiovascular diseases (CVD) are still a leading cause of mortality and morbidity around the world. According to World Health Organization (WHO), in 2019 about 17.9 million people died from CVDs, which constitute 32% of all deaths (1). The etiology of CVD is heterogenous and arises from an interplay between genetic components (non-modifiable risk factors) and environmental/lifestyle determinants (usually modifiable) which can lead to deterioration of endothelial structure and function as well as atheromatous plaque formation as an early stage in the development of atherosclerotic CVD (2). Subsequent artery narrowing or occlusion remains the most crucial element of developing CVD, increasing the risk of cardiovascular events such as stroke, myocardial infarction, heart failure, and peripheral artery disease, as well as renal dysfunction, and cognitive impairment (3, 4).

### Environmental noise in the spectrum of cardiovascular risk factors

To estimate a 10-year risk of fatal and non-fatal CVD events in European populations, the Systematic COronary Risk Evaluation (SCORE2) algorithm is widely used. However, this score includes only traditional risk factors, such as age, sex, blood pressure, plasma cholesterol, and cigarette smoking (5, 6). Other well-established risk factors like diabetes mellitus, unhealthy diet, sedentary lifestyle, obesity, and alcohol consumption are included (1). In addition, recent studies recognize many underlying determinants of CVDs that arise mostly from globalization and urbanization which include exposure to air and soil pollution as well as above-threshold noise levels (5, 7). Noise is defined as an unpleasant or harmful sound. The most common environmental noise sources studied are those produced by transportation (road traffic, railway, and aircraft) and industry (8, 9). It can affect human health depending on the characteristics of the sound and time of exposure, as well as subjective perception described as annoyance (10). The noise effect on the cardiovascular system is associated with chronic low-level exposure, which dysregulates homeostasis through autonomic nervous and endocrine system arousal (11). Recent animal studies, as well as observational studies on humans, show that especially night-time noise is critical to endothelial dysfunction development, which indicates that sleep deprivation and fragmentation due to noise annoyance are important mechanisms of stress-related damage (12, 13). Noise activates the pituitary–adrenal–cortical axis and the sympathetic–adrenal–medullary axis. It also provokes fluctuations of stress-related hormones including epinephrine, norepinephrine, and cortisol (14). These stress responses induce cardiovascular and cerebral oxidative stress and inflammation (15). Sustained, chronic reaction to stress increases and accelerates the risk of developing CVD such as arteriosclerosis, hypertension, and ischemic heart disease (16).

### Environmental noise regulations

The European noise policy has been developed and harmonized for over two decades. In 1996, the Commission of the European Communities enlightened the problem of increased noise exposure due to urbanization and recognized it as the main environmental problem in Europe (17). Noise emission limits have been successively laid down for most of the urban-related noise sources according to the Directive of the European Parliament and the Council published in 2002 (18). Especially aircraft noise had been taken into consideration, as this noise is characterized by the highest annoyance as compared with other transportation noise sources (19). Annoyance is a term used in order to describe subjective negative feelings such as displeasure or irritation caused by noise. It can be measured semi-quantitatively through dedicated questionnaires (20). There is a level-dependent relationship between noise and annoyance, however, it is different for different noise sources. Aircraft noise has been reported to be more annoying than other transportation noises at the same noise level, which was highlighted by European Agency Report (20, 21).

Sound exposure levels are expressed in decibels (dB), and to assess chronic exposure to noise, long-term indices are in use (22). The most commonly used are average sound pressure level over all day–evening–night periods in a year (Lden) and average sound level over all night periods in a year (Lnight) (22, 23). With these standardized indicators, it is possible to create acoustic maps containing noise levels to assess the number of people exposed to different levels of noise, which can be harmonized for all EU Member States (9). The WHO Guideline Development Group recommends reducing aircraft noise below 40 dB Lnight as the noise above this level is linked with health adverse effects (9). Noise effect on human health can be associated with auditory, or direct consequences, as well as non-auditory (indirect). CVDs develop as an indirect noise effect and result from chronic exposure (24).

In this mini-review, we aimed to discuss the results of our studies, focused on aircraft noise exposure consequences for CV systems, with other currently published study results in this field with regard to the WHO Environmental Noise Guidelines for the European Region.

## Cardiovascular consequences of aircraft noise exposure

Rojek et al. conducted a cross-sectional study between 2015 and 2016, among participants exposed to high (above 60 dB Lden) and low (below 55 dB Lden) aircraft noise levels to assess the impact of long-term exposure to aircraft noise on blood pressure (BP), prevalence of arterial hypertension, and indices of asymptomatic organ damage (10). The authors revealed that long-term aircraft noise exposure was associated with higher blood pressure indices, that is, higher office and night-time diastolic blood pressure (DBP). Moreover, in the group exposed to higher aircraft noise levels, more advanced hypertension-related organ damages were observed, that is, increased pulse wave velocity (PWV), the measure of arterial stiffness, and lower values of tissue Doppler-derived early diastolic mitral annulus velocity (E')—representing an early stage of diastolic left ventricular dysfunction (10, 25, 26). Of note, higher office and night-time DBP, PWV, and E' values were explicitly observed in exposed normotensive participants. Moreover, PWV in aircraft noise-exposed normotensive participants was equal to that of two decades older unexposed normotensive participants and was significantly associated with noise annoyance. Numerous studies focused on the relation between environmental noise exposure and hypertension prevalence or BP increase (27, 28). While data on subclinical organ damage are scarce, Foraster et al. in the SAPALDIA study revealed that long-term railway noise exposure during the night and the number of night-time noise events related to road traffic were associated with arterial stiffness (29). In a human field study, Schmidt et al. (30) observed an increase in arterial stiffness assessed by the carotid–femoral transit time (a component used in the calculation of PWV) as a consequence of nocturnal exposition to aircraft noise in healthy individuals (30). They revealed also an increased release of catecholamines after noise exposure suggesting the involvement of sympathetic nervous system activation by stress mechanisms (30). Moreover, noise exposure impaired endothelial function in patients with or at high risk for coronary artery disease, which was pronounced in these patients compared to healthy subjects (31). Münzel et al. (16) extensively described the pathophysiological mechanisms connecting noise exposure with stress reaction neuro-humoral activation, and hemodynamic and metabolic consequences (16).

The importance of the SAPALDIA study resulted from a large sample size (*N* = 2775), while the strength of the Rojek study results from a very detailed and wide assessment of the blood pressure phenotype [including 24-h ambulatory blood pressure monitoring (ABPM)] and the analysis of three different indices of cardiovascular damage: intima–media thickness, arterial stiffness, and echocardiography. Measured parameters of organ damage have a crucial prognostic value to assess the risk of cardiovascular complications and mortality (16).

In a subsequent study from the same group of authors (8), the impact of chronic night-time exposure to aircraft noise on BP profile, sleep disturbances, and annoyance was assessed in individuals without hypertension (8). The participants were divided into two groups regarding their exposure to night-time aircraft noise. Exposed individuals were selected from an area exposed to noise exceeding 50 dB of Ln, while the unexposed group lived in the village exposed to noise below 45 dB. Insomnia was assessed by means of the Athens Insomnia Scale (AIS) and was defined as AIS above six points (32). Individuals living in the night noise-exposed area were characterized by a 2-fold higher insomnia prevalence than those living in the low noise-exposed area (33 vs. 16%, *p* = 0.046) and reached higher scores in AIS. As a consequence of sleep disturbances induced by noise, the authors observed higher diastolic BP at night and higher office DBP in exposed individuals compared to those living in a low noise exposure area (8).

A greater prevalence of insomnia and impaired sleep quality was also reported among individuals living near Orio al Serio International Airport by Carugno et al. (33). In contrast to the results obtained by Rojek et al. and Carugno et al. (8, 33) found no differences in BP between aircraft noise-exposed and unexposed subjects. A potential explanation may include the difference in the noise level threshold and type of indices between these studies (Lnight in Rojek study, Lden in Carugno study) which may result in a less pronounced contrast between exposed and unexposed subjects.

The results obtained by Rojek regarding the influence of aircraft noise on noise-exposed residents are largely in agreement with the results obtained by Schmidt et al. (34). In this experiment, 70 individuals with cardiovascular risk factors or established cardiovascular disease were subjected to three different scenarios. The control scenario comprised no noise exposure (regular background noise in the sleeping room of the participants), and the noise scenario nights comprised 60 and 120 noise events, respectively. In both noise scenario nights, subjects were exposed to similar equal average sound pressure levels (Leq). In all experimental conditions, polygraphy recordings, echocardiography, measurement of flow-mediated dilation (FMD), and blood sample analysis were performed. Questionnaires regarding sleep quality and annoyance were also collected (34). The study demonstrated a worsening of vascular function after noise-exposed nights compared to control nights. Furthermore, worsening of left ventricular diastolic function (*p* = 0.043) displayed by an increased ratio between early mitral inflow velocity and mitral annular early diastolic velocity (E/E') was observed in both night noise scenarios. Plasma analysis after noise exposure (120 events) revealed a decrease in follistatin (FS) (*p* = 0.016), glyoxalase I (GLO1) (*p* = 0.044), and angiotensin-converting enzyme 2 (ACE2) (*p* = 0.032). These findings prove that night-time noise is a factor in deteriorating endothelial function, increasing the risk of developing fibrosis, and increasing metabolic stress through impaired detoxification of reactive aldehydes. There were significant (p<0.001) differences in FMD between scenarios with noise events (7.27 ± 3.21% for Noise 60 and 7.21 ± 3.58% for Noise 120) and the control night (10.02 ± 3.75%). This finding proved that short-time noise exposure leads to endothelial dysfunction. The study did not show any statistically significant differences in BP between observed individuals (34). However, the short-term exposure to noise led to numerous vascular and biochemical changes which may explain the long-term noise effects on BP changes as observed by Rojek et al. (8).

## Cardiovascular consequences of noise reduction

Although the adverse effects of aircraft noise on the cardiovascular system are well-established, less is known about the potential reversibility of these changes after noise reduction, which is suggested by regulatory measures. Evidence on this issue came from Wojciechowska et al. by following up with the individual in 2020 from the Rojek et al. (35) study. After a follow-up of 5.5 years, the authors evaluated the impact of a sudden decline in air traffic for about 4 months which took place due to the COVID-19 lockdown. As a result, the average aircraft noise level decreased from 61.7 to 47 dB during the day and 55.4 to 43.4 dB during the night period in the region previously marked as exposed to aircraft noise in 2015. Therefore, both investigated groups were exposed to similar levels of aircraft noise. The study was conducted with the same protocol as the Rojek study in 2015. ABPM, PWV, and echocardiographic measurements were performed, and questionnaires regarding annoyance and insomnia were collected. The prevalence of hypertension in the exposed group increased in the follow-up. This is consistent with other studies, proving the long-lasting effects of aircraft noise exposure on hypertension risk (36, 37). Due to the aging of the cohort, an increase in arterial stiffness was expected in both the exposed and unexposed groups. In both groups, the decline in PWV was observed; however, the decrease in PWV during follow-up assessment was more pronounced in the exposed group (mean 10.2 vs. 8.8 m/s at follow-up, *p* = 0.001) as a result of noise level decline (35). A similar relationship was observed concerning noise-induced annoyance. In the exposed group, annoyance significantly decreased in this period (*p* = 0.006) but was still more pronounced compared to the unexposed group. During the follow-up visit, lower BP values were observed in both groups regardless of their noise exposure, although a decrease in office, central BP, and night-time DBP was significantly dipper in the group of previously exposed individuals (35). Moreover, after calculating the estimated BP values at a 5.5-year follow-up and comparing it with actually measured BP values in the individuals from the exposed group without antihypertensive treatment, the observed office DBP was significantly (*p* = 0.048) lower than the expected DBP. There were no such differences in the unexposed group (35). Both reductions in noise-inducted annoyance and drop in BP during the follow-up visit are factors that could explain the decrease in arterial stiffness. The fact that reduction in noise exposure resulted in more pronounced PWV changes than BP changes is consistent with the results of the study by Schmidt et al. (30). The study by Wojciechowska et al. revealed that even the short-time reduction of aircraft noise can not only reverse the unfavorable long-term effect on BP and arterial stiffness. Another important finding is that the decline in aircraft noise during the pandemic restores the natural relation between PWV and age. This relation was previously blunted by the noise influence, that is, there was no increase in PWV with age (35).

Unlike other cardiovascular risk factors, such as tobacco intake or unhealthy lifestyle, noise exposure cannot be managed on the outpatient clinic level, but rather by guidelines followed by regulations on local and systemic levels. Hahad et al. (28) compared the publication of Wojciechowska et al. (35) with previous reports regarding the influence of noise on human health, raising the importance of treating noise as a psychosocial stressor leading to annoyance, which induces a cascade of neuroendocrine changes resulting in elevated blood pressure and increased stress hormone levels and heart rate (28). These processes contribute to the development of cardiovascular diseases, which was widely discussed in another manuscript by Hahad et al. (38). These results show the inter-relationship between noise, annoyance, and arterial stiffness and provide evidence that eliminating noise as the underlying cause also reduces annoyance and arterial stiffness (35). Consequently, long-term exposure to aircraft noise *via* different mechanisms with a leading role in endothelial dysfunction is accompanied by a higher prevalence of hypertension (15). The most important finding of the cohort study by Wojciechowska et al. (35) is the reversible character of noise effect on subclinical organ damage, even with the short-term decrease in air traffic. This shows the importance of noise level restriction implementations, consistent with WHO recommendations, as an important aspect of managing public health.

## Discussion

**Figure 1:**
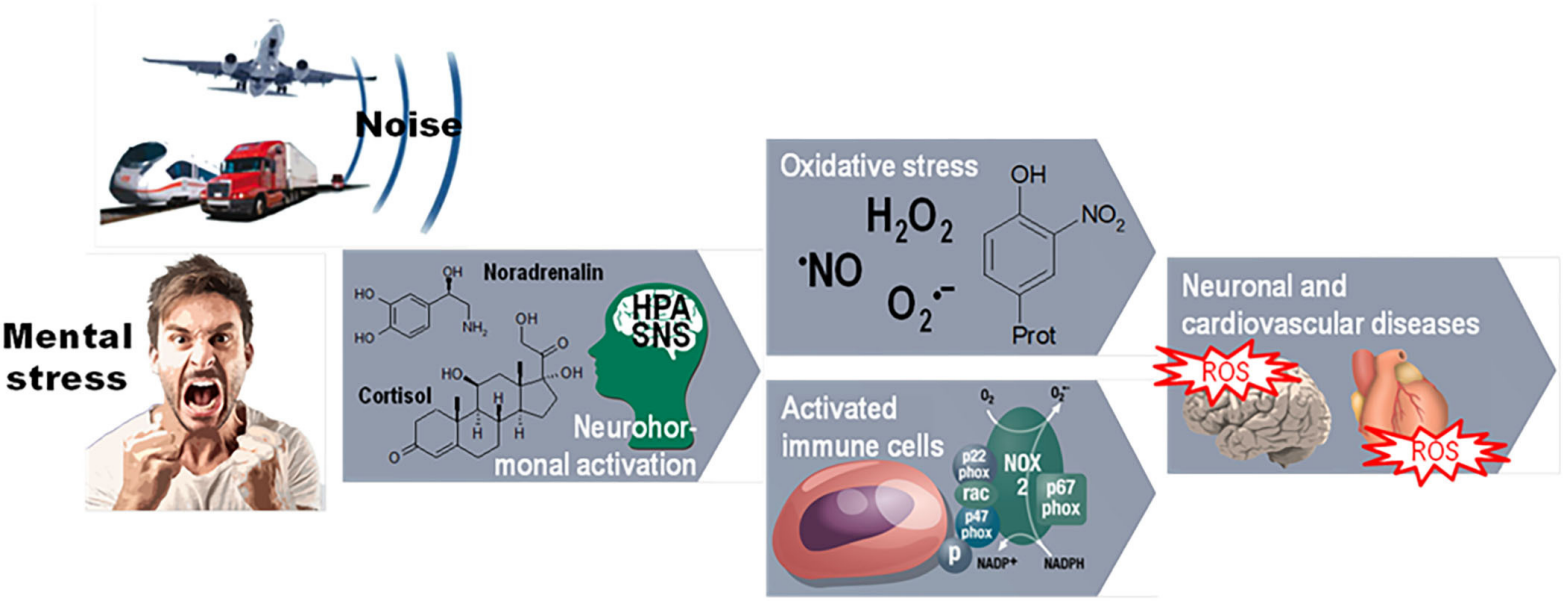
Pathways of environmental noise action on cardiovascular disorders through mental stress and neurohormonal, immune, and oxidative stress mechanisms. The environmental risk factors noise exposure and mental stress cause a primary stress reaction, mediated either by the hypothalamic–pituitary–adrenal (HPA) axis with subsequent cortisol release or by activation of the sympathetic nervous system (SNS) with subsequent catecholamine formation. These stress reactions activate inflammatory or oxidative stress pathways which can stimulate each other and, together with stress hormones, vasoconstrictors, and alterations of gene expression, contribute to the classical risk factors such as hypertension and atherosclerosis leading to neuronal, cardiovascular, and metabolic diseases. Reused from Daiber A, Kröller-Schön S, Frenis K, Oelze M, Kalinovic S, Vujacic-Mirski K, et al. Environmental noise induces the release of stress hormones and inflammatory signaling molecules leading to oxidative stress and vascular dysfunction-Signatures of the internal exposome. Biofactors. 2019 Jul;45(4):495–506. doi: 10.1002/biof.1506 with permission.

Exposition to aircraft noise increases the prevalence of insomnia, evoked annoyance, sleep disorders, and subsequently early functional and structural changes in the endocrine and cardiovascular system such as an increase in stress hormones, oxidative stress, endothelial dysfunction, and arterial stiffness (Figure 1). At this stage, those changes are still reversible. However, the late consequences of long-lasting noise exposure, that is, established cardiovascular diseases, are not only indisputably confirmed by numerous epidemiological studies but also are irreversible. The only way of mitigating noise-induced health burden is to systematically reduce the noise level as recommended by the WHO (9). Even though environmental noise has been recognized as a burden of public health almost two decades ago, there is still an area for action to improve noise regulations in Europe.

## Author contributions

JB, WW, MRo, OH, AD, TM, and MRa: gathering, analyzing, interpreting data for the work, drafting, and revising the manuscript. All authors contributed to the article and approved the submitted version.

## Funding

This work was funded by Jagiellonian University Collegium Medicum.

## Conflict of interest

The authors declare that the research was conducted in the absence of any commercial or financial relationships that could be construed as a potential conflict of interest.

## Publisher's note

All claims expressed in this article are solely those of the authors and do not necessarily represent those of their affiliated organizations, or those of the publisher, the editors and the reviewers. Any product that may be evaluated in this article, or claim that may be made by its manufacturer, is not guaranteed or endorsed by the publisher.

## Abbreviations

ABPM: 24-h ambulatory blood pressure monitoring
ACE2: glyoxalase I (GLO1) angiotensin-converting enzyme 2
AIS: Athens Insomnia Scale
BP: blood pressure
COVID-19: coronavirus disease 2019 caused by SARS-CoV-2
CVD: cardiovascular disease
dB: decibels
DBP: diastolic blood pressure
E/E': ratio between early mitral inflow velocity and mitral annular early diastolic velocity
E': tissue Doppler-derived early diastolic mitral annulus velocity
EU: European Union
FMD: flow-mediated dilation
FS: follistatin
GLO1: glyoxalase I
HPA: hypothalamic–pituitary–adrenal axis
LA, A, HA: little annoyed, annoyed, highly annoyed
Lden: average sound pressure level over all day–evening–night periods in a year
Leq: equal average sound pressure level
Lnight: average sound level over all night periods in a year
PWV: pulse wave velocity
SCORE2: Systematic COronary Risk Evaluation
SNS: sympathetic nervous system
WHO: World Health Organization.
